# Genomics of Evolutionary Novelty in Hybrids and Polyploids

**DOI:** 10.3389/fgene.2020.00792

**Published:** 2020-07-28

**Authors:** Gonzalo Nieto Feliner, Josep Casacuberta, Jonathan F. Wendel

**Affiliations:** ^1^Department of Biodiversity and Conservation, Real Jardín Botánico, CSIC, Madrid, Spain; ^2^Center for Research in Agricultural Genomics, CRAG (CSIC-IRTA-UAB-UB), Barcelona, Spain; ^3^Department of Ecology, Evolution, and Organismal Biology, Iowa State University, Ames, IA, United States

**Keywords:** adaptation, allopolyploidy, gene and genome duplication, transposable elements, hybridization, phenotypic novelty, radiation lag-time model

## Abstract

It has long been recognized that hybridization and polyploidy are prominent processes in plant evolution. Although classically recognized as significant in speciation and adaptation, recognition of the importance of interspecific gene flow has dramatically increased during the genomics era, concomitant with an unending flood of empirical examples, with or without genome doubling. Interspecific gene flow is thus increasingly thought to lead to evolutionary innovation and diversification, via adaptive introgression, homoploid hybrid speciation and allopolyploid speciation. Less well understood, however, are the suite of genetic and genomic mechanisms set in motion by the merger of differentiated genomes, and the temporal scale over which recombinational complexity mediated by gene flow might be expressed and exposed to natural selection. We focus on these issues here, considering the types of molecular genetic and genomic processes that might be set in motion by the saltational event of genome merger between two diverged species, either with or without genome doubling, and how these various processes can contribute to novel phenotypes. Genetic mechanisms include the infusion of new alleles and the genesis of novel structural variation including translocations and inversions, homoeologous exchanges, transposable element mobilization and novel insertional effects, presence-absence variation and copy number variation. Polyploidy generates massive transcriptomic and regulatory alteration, presumably set in motion by disrupted stoichiometries of regulatory factors, small RNAs and other genome interactions that cascade from single-gene expression change up through entire networks of transformed regulatory modules. We highlight both these novel combinatorial possibilities and the range of temporal scales over which such complexity might be generated, and thus exposed to natural selection and drift.

## Introduction

One of the remarkable realizations of the genomics era is that hybridization—crosses between individuals from populations that are distinguishable on the basis of one or more heritable characters (Harrison, 1990)—and interspecific gene flow—the successful movement of genes among populations (Ellstrand, 2014)—are far more prevalent than previously recognized. Genomic data from a wide range of model and non-model organisms have both confirmed and greatly extended the pre-existing notion that natural hybridization is a frequent phenomenon in the living world, and not only in plants (Mallet, 2005; Soltis and Soltis, 2009; Green et al., 2010; Fontaine et al., 2015; Leducq et al., 2016; Elgvin et al., 2017; Meier et al., 2017; Lamichhaney et al., 2018), and that it often leads to interspecific gene flow. The observation that hybridization is associated with allopolyploid speciation has long been recognized, and in fact this became fundamental in plant evolutionary thinking, with the original evidence primarily consisting of cytological observations that peaked in the 1970s (Stebbins, 1940, 1971; Grant, 1981). The advent of molecular markers magnified this interest, stimulating research into polyploidy across the phylogenetic spectrum of angiosperms and using both natural and synthetic allopolyploids (reviewed in, e.g., Soltis and Soltis, 2012; Soltis et al., 2015, 2016; Van de Peer et al., 2017). This interest continues apace today, as evidenced by the present special issue of *Frontiers*.

Among the more surprising insights to emerge from this research is that the genomes of *all* modern angiosperms contain vestiges of multiple past rounds of polyploidy (Jiao et al., 2011; Soltis et al., 2016; Leebens-Mack et al., 2019; but see Ruprecht et al., 2017a), some ancient and in many cases some quite recent, with each event superimposed on the genomic remnants of earlier rounds of doubling. A second realization is that each whole genome doubling (WGD) event has been followed by incompletely understood genome fractionation processes (Wendel, 2015; Soltis et al., 2016; Bird et al., 2018; Cheng et al., 2018; Wendel et al., 2018), as well as myriad immediate and longer-term responses to genome merger and doubling at the genomic, expression, and cellular levels (Yoo et al., 2014; Wendel, 2015; Soltis et al., 2016; Sharbrough et al., 2017; Doyle and Coate, 2019). Thus, the architecture of modern plant genomes reflects, in part, the residuals from the superimposed joint action of WGD and fractionation, these twin processes encompassing the “wondrous cycles of polyploidy” (Wendel, 2015).

One consequence of the pervasiveness of polyploidy and its dynamism over time is the widely held view that whole-genome doubling plays an important role in generating phenotypic novelty. This topic has been the subject of speculation for decades (Levin, 1983; Soltis, 2013; Soltis P. S. et al., 2014; Vanneste et al., 2014; Edger et al., 2015; Tank et al., 2015; Van de Peer et al., 2017), but to date the number of cases where polyploidy itself has been convincingly connected to specific phenotypic innovations remains relatively small. Part of the challenge in demonstrating this connection is that adaptation and diversification take place over a diverse spectrum of time-scales, as do the various genomic diversification and fractionation processes set in motion by polyploidy.

In addition to hybridization *with* WGD (allopolyploidy), evidence abounds for the occurrence of homoploid hybridization, that is, hybridization and gene flow *without* WGD (Mallet, 2007; Pennisi, 2016; Runemark et al., 2019). Three decades of molecular phylogenetic studies (e.g., Sang et al., 1995; Barrier et al., 1999; Blanco-Pastor et al., 2012; García et al., 2017; Marques et al., 2017; Folk et al., 2018) and more recent genomic scrutiny (Baack and Rieseberg, 2007; Twyford and Ennos, 2012; Abbott et al., 2016; Payseur and Rieseberg, 2016) have revealed numerous hybrid lineages across the living world. Ever since hybridization and introgression started to be inferred from incongruence between gene-trees, it has been realized that an alternative neutral process—incomplete lineage sorting (ILS) or deep coalescence—could lead to similar phylogenetic patterns. Favored by large populations sizes and short speciation times, the occurrence of ILS is still often inferred whenever evidence for introgression is lacking. However, a number of methodological approaches, the most widely used of which is the ABBA-BABA test, are now available to tease apart the two phenomena (Joly et al., 2009; Green et al., 2010; Blanco-Pastor et al., 2012). What remains a matter of some debate is how often hybridization plays a creative role in evolution, or phrased alternatively, whether the advantages accrued from natural hybridization are responsible for the pervasiveness of this phenomenon. The influential ideas of Mayr (1963) that hybridization was an evolutionary dead-end began to be challenged after breakthrough discoveries in birds and insects (Mavárez and Linares, 2008; Grant and Grant, 2009, 2017; Salazar et al., 2010), but the relative importance of hybridization in lineage diversification remains a subject of active debate. Some posit that hybridization, even if frequent, is likely transient in genomes and thus of little evolutionary relevance (Barton, 2013; Servedio et al., 2013); according to this view, merged genomes mostly evolve in the direction of purging incompatibilities (Schumer et al., 2016, 2018). The opposite view, that natural hybridization may contribute positively to adaptation, differentiation and speciation, is embraced by many empirically oriented evolutionary biologists (Rieseberg, 1991; Arnold, 1993; Wang et al., 2001; Mallet, 2007; Abbott et al., 2010, 2013; Butlin and Ritchie, 2013; Sætre, 2013; Soltis, 2013; Yakimowski and Rieseberg, 2014; Grant and Grant, 2017; Nieto Feliner et al., 2017; Ottenburghs, 2018; Wagner, 2018), impressed as they are by the ever-increasing number of discoveries of gene-tree conflict in datasets. In part, these two views are fueled by the contrast between the burgeoning number of lineages that are unveiled by molecular phylogenetic studies to have a hybrid ancestry and the tiny fraction of cases in which we understand how hybridization may have led to adaptation (and/or phenotypically relevant drift) and diversification (Schumer et al., 2014). We suspect that this scarcity of well-understood examples reflects both insufficiency in our understanding of the genetic bases of adaptive traits and their inherent diversity and context dependency, as well as the temporal disconnect between hybridization events (ancient and recent) and adaptation to ecological conditions that may no longer be present.

Allopolyploidization and homoploid hybrid speciation, of course, comprise just two of the many possible evolutionary outcomes of interspecific genetic exchange (Runemark et al., 2019). Others include reinforcement (Hopkins, 2013), genetic assimilation (Levin et al., 1996; Ehrenreich and Pfennig, 2016), formation of various kinds of hybrid zones (Barton and Hewitt, 1985; Harrison, 1993; Abbott, 2017), and adaptive or neutral introgression (Rieseberg and Wendel, 1993; Heliconius Genome Consortium, 2012; Schmickl et al., 2017; Suarez-Gonzalez et al., 2018b; Edelman et al., 2019). These many possibilities serve to illustrate both the prevalence and complexity of the outcomes of secondary contact among divergent lineages, and additional, seemingly unlikely possibilities continue to emerge. For instance, inter-ploidy gene flow between lineages that “should be” reproductively isolated turns out to characterize the evolution of many allopolyploid lineages or diploid-allopolyploid complexes (Grant, 1981; Zohren et al., 2016; Hohmann and Koch, 2017; Marburger et al., 2019; Monnahan et al., 2019).

There are also connections between hybrid zones and homoploid hybrid speciation (Hodges et al., 1996), and between adaptive introgression and homoploid hybrid speciation (Brower, 2013). In fact, some authors propose that these outcomes of hybridization represent different stages of a continuum of speciation (Seehausen et al., 2014; Lowry and Gould, 2016; Roux C. et al., 2016). This notion illustrates how hybridization usually occurs in a complex spatial and temporal context (Abbott et al., 2013; Sætre, 2013). Multiple different factors, such as levels of divergence and ecological opportunity, may determine whether raw genetic variance introduced by hybridization—two to three orders of magnitude greater than that introduced by mutation, according to Grant and Grant (1994)—facilitates adaptive divergence or contributes to evolutionary novelty. That the ultimate outcomes of natural hybridization and allopolyploidy depend on numerous interacting factors sieved by selection over various timescales and ecological contexts makes predictions extremely difficult (Butlin and Ritchie, 2013), and favors expectations that somehow incorporate stochasticity, e.g., an evolutionary novelty hybrid zone model (Arnold, 1997). Examples of this stochasticity in the short term includes hybrid unviability of some genotypes even in F_1_ hybrids between conspecific genotypes of an inbreeding species (Bomblies and Weigel, 2007) and post-F_1_ hybrids between species due to Bateson-Dobzhansky-Muller (BDM) incompatibilities and/or breakdown of coadaptive gene complexes following recombination (Christe et al., 2016).

Given the numerous avenues by which genetic exchanges may occur between differentiated genomes, we thought it timely to consider the question of how this merger might ultimately lead to novel phenotypes and adaptation. We leave aside the classic but still debated topic of heterosis, which refers to the process by which hybrids, including allopolyploids, may exhibit greater biomass, speed of development, and fertility than both parents (Birchler et al., 2010; Hochholdinger and Baldauf, 2018). At the outset, hybridization leads to infusion of new genetic material, which is either rapidly removed by selection and/or drift, or partially removed, leaving behind a transformed genome. But in most cases, even involving iconic examples such as *Iris* (Martin et al., 2006), *Helianthus* (Rieseberg et al., 2003), and *Populus* (Suarez-Gonzalez et al., 2016, 2018b) the specific identity and connections between introgressed material and adaptive phenotypes remains elusive or at least incompletely defined. In this light, and perhaps as a form of foreshadowing, we therefore consider the spectrum of novel molecular genetic and genomic processes that may be set in motion by the saltational genomic shock of merger, both with and without genome doubling. Our focus is on the possible selective advantages of hybridized genomes that lead to adaptation, phenotypic novelty, and diversification, as opposed to simply transient effects. Using examples mostly from plants we highlight genetic and genomic consequences of genome merger that create potentially adaptive phenotypes. Our intention is not to be comprehensive nor encyclopedic, but instead to present an overview of the possible mechanisms by which new phenotypes may arise as a result of interspecific gene flow. As an organizational framework, we arrange these effects into three categories, noting that these are not mutually exclusive and often occur in concert: (1) responses at the genetic and genomic level, such as structural diversity or copy number variation; (2) responses at the gene expression and regulatory level, such as neo- and subfunctionalization of duplicated loci; and (3) responses at combinatorial genomic and expression-levels, such as cytonuclear interactions or transposable element activity.

## Responses to Genome Merger at the Genetic and Genomic Level

### Genic Introgression

**FIGURE 1:**
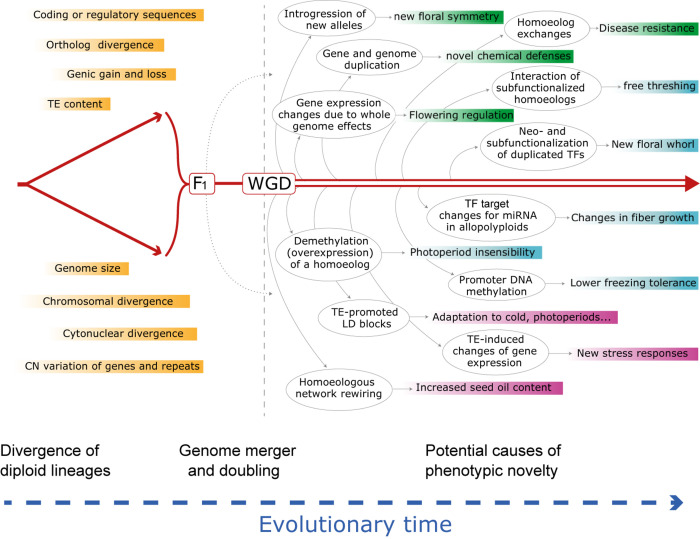
Schematic evolutionary diagram depicting changes accompanying divergence of two diploid lineages, followed by phenomena enabled by or set in motion by hybridization and genome doubling, either separately or together. Shown is a sampling of genetic and genomic mechanisms subsequent to hybridization and an allopolyploid event that could, over time (arrow at bottom), lead to novel adaptation and phenotypes. Many of these phenomena and consequences may be latent for hundreds to millions of years. Box colors indicate categories of change in the order discussed in the text: responses at the genomic and genetic level (green); responses at the gene expression and regulatory level (cyan); responses at the combinatorial genomic and expression levels (mauve). See text for elaboration of these and many other examples. CN, copy number; HE, homoeologous exchange; LD, linkage disequilibrium; miRNA, microRNA; TE, transposable element; TF, transcription factor; WGD, whole genome duplication. At present the relative importance and prevalence of these phenomena following hybridization vs. polyploidy is unknown, but in many cases the two organismal-level processes independently set in motion at least some of the illustrated genomic responses.

Plant genomes vary enormously in virtually every feature used to describe their composition or “suite of residents,” including, from the smallest scale to the largest, nucleotide composition, gene and regulatory sequences, genic content and copy numbers, repetitive sequences and transposable element content, chromosome numbers, genome size (Wendel et al., 2016) and a spectrum of epigenetic features. As populations and species diverge, so will their genomes, though not necessarily monotonically across categories of genomic change nor homogeneously among lineages. Nonetheless, the divergence of lineages is inevitably associated with the accumulation of multiple forms of mutational differences, again, small to large. At the simplest level, divergence is associated with changes in gene sequence, either in coding or regulatory sequences, and thus genomic reunions associated with hybridization and/or WGD may lead to new genic contexts with possible effects on selectively relevant phenotypes (Figure 1).

In principle, hybridization-induced infusion of diverged or novel genes might be considered the most straightforward form of evolutionarily relevant introgression to detect following hybridization, as it is a straightforward matter now to assay gene sequences. Accordingly, it is not surprising to find recent examples of adaptation or at least phenotypic novelty caused by genic introgression. An elegant early example was from *Senecio* (Kim et al., 2008), who showed that introgression of the RAY locus from the diploid hybrid species *S. squalidus* into the tetraploid *S. vulgaris* causes the formation of bilaterally symmetrical flowers (ray florets) on the periphery of the heads that otherwise are discoid. The introgressed RAY locus comprises a cluster of cycloidea-like genes, which encode DNA-binding proteins known to cause asymmetry in *Antirrhinum* (Luo et al., 1996). One of the more remarkable features of the introgressed RAY locus story is that the gene flow surmounted a seemingly improbable reproductive barrier, from diploid to tetraploid. How this occurs is unclear, but interploidal gene flow is a recurring theme (see below). An added notable dimension to this example is that the gene flow apparently is adaptive, as ray florets promote outcrossing and thereby infuse genetic variation into the otherwise selfing *S. vulgaris* (Kim et al., 2008).

A second beautiful example of inter-ploidal gene flow and adaptation to the polyploid condition is the paper (Marburger et al., 2019) on diploid and autopolyploid *Arabidopsis arenosa* and *A. lyrata* in Europe. Both species have diploid and autopolyploid populations and experience at least occasional diploid-tetraploid gene flow. By resequencing 92 individuals from 30 populations collected from a broad range of both species, Marburger et al. (2019) demonstrate that interspecific introgression has occurred bidirectionally, and that some *A. arenosa* introgression peaks into *A. lyrata* are both narrow and associated with strong signatures of selection. Remarkably, these small regions of interspecific introgression include key genes known to be important in stabilizing meiosis following WGD, suggesting that adaptation to polyploidy was mediated by interspecific gene flow. A fascinating twist on this story is that the *A. arenosa* alleles introgressed into *A. lyrata* are posited to have been favored because WGD in the former species is older than in the latter. Thus, its alleles at meiosis-stabilizing genes are better adapted to the tetraploid condition than the more naïve and native *A. lyrata* genes, and so selection has favored their replacement.

The preceding examples of adaptive genic introgression represent the unusual cases where adaptation has been at least arguably causally connected to specific genes. Far more numerous are examples where genomic evidence for adaptive introgression is convincing, but either the responsible genes have not been identified or they have not been directly linked to phenotypes that are unequivocally connected to adaptation (Suarez-Gonzalez et al., 2016, 2018a; Hübner et al., 2019; Janzen et al., 2019; Mitchell et al., 2019). A recent case in point is for cultivated sunflowers (Hübner et al., 2019), where genomic resequencing of about 400 cultivated lines, Native American landraces and wild accessions from 11 wild species demonstrated that 1.5% of the genes in cultivated sunflower arose via interspecific introgression from wild species. Many of these genes are connected to biotic resistance such as downy mildew resistance, implicating selection for disease resistance as being responsible for adaptive interspecific gene flow. Similar examples of either intentional or unintentional adaptive introgression are common in our major cultivated crops (Janzen et al., 2019).

The preceding examples all involve either introgression between closely related congeners or wild-domesticated comparisons. In these instances the temporal window for detecting adaptive introgression may be maximized relative to other scales of divergence, in that as the latter increases, along with time since divergence, there is less certainty with respect to the relevant ecology (and hence insight into selective pressure). An interesting recent example in this respect is from Tibetan *Cupressus* (Ma et al., 2019), where a variety of transcriptomic and population genetic tools were used to demonstrate that adaptation to colder and drier environments in one species was enabled via introgression from a second species, perhaps 200,000 years ago. Detecting the footprints of selection will be more difficult, however, as equilibrium is restored following selective sweeps.

Challenges in detecting adaptive introgression are not just restricted to older events, because even recent adaptive introgression may be difficult to distinguish from other causes of patterns of variation. Clinal variation across environmental gradients, for example, may arise from local differentiation, or, as in the case of *Cupressus* cited above (Ma et al., 2019), from asymmetric interspecific introgression (see also, e.g., Welch and Rieseberg, 2002; Rieseberg et al., 2003; Arnold et al., 2010; Scascitelli et al., 2010; Whitney et al., 2010; Leroy et al., 2020). A powerful approach for distinguishing adaptive introgression from other sources of variation entails the combined use of genome-wide tools, now accessible for most non-model organisms, with demographic and population genetic modeling (Pease et al., 2016; Aeschbacher et al., 2017; Martin and Jiggins, 2017; Ma et al., 2019). Genome-wide approaches also provide insight into key questions such as whether introgression is scattered or localized across the genome, how it has been shaped by selection (Suarez-Gonzalez et al., 2016), and whether adaptively introgressed alleles had diverged in the donor species (Parchman et al., 2013; Bay and Ruegg, 2017; Leroy et al., 2020). These considerations are finding increasing utility in the rescue of genetically impoverished or threatened species (Hamilton and Miller, 2016).

These many challenges associated with the passage of time, historical ecological inference, and interpretation of patterns of genetic diversity also apply to our understanding of adaptation following ancient episodes of polyploidy. In most cases, it is unknown whether older WGD events involved autopolyploidy or allopolyploidy, and thus even though it might be clear that there are functionally divergent homoeologs (including subfunctionalized and neofunctionalized), it is not at all evident whether this divergence represents evolution at the polyploid level or whether this reflects merger of pre-existing differences. Comparative genomics often, however, yields important clues, as in the example of the butterfly (Pieridae)-glucosinolate “arms race” in the Brassicales (Edger et al., 2015), where gene and genome duplication is implicated in novel chemical defenses in the plants as well as countermeasures in the butterflies. Similarly suggestive evidence is common in other plant groups (Sato et al., 2012; Vanneste et al., 2014; Lohaus and Van de Peer, 2016), and certain classes of genes and transcription factors involved in stress responses have been found to be repeatedly preferentially retained following WGDs in 25 different angiosperm lineages (Wu et al., 2019). These examples collectively provide tantalizing evidence which implicate, but do not prove, that WGD was responsible for evolutionary specializations or adaptations.

### Structural Diversity

Merger of differentiated genomes leads not only to transfer of genes but also to the incorporation of structural variants in a novel genomic context. Structural variants (SVs) include differences in copy number (copy number variation; CNV) of genes and repeats, presence-absence variation (PAV), various forms of chromosomal change such as inversions (Huang and Rieseberg, 2020) and translocations, and homoeologous exchanges (HEs; see Mason and Wendel, this issue). In fact, the two or more co-resident genomes of allopolyploids almost certainly contain structural variations, which have been shown to accumulate in all plant genomes studied (Saxena et al., 2014; Fuentes et al., 2019; Gabur et al., 2019; Schiessl et al., 2019). Thus, divergence of two diploids is accompanied by the natural accumulation of structural differences, which then become combined in a common nucleus during allopolyploidization.

The scale of structural variation within and among plant species represents an extraordinary discovery of the genomics era; that is, rather than SVs being rare, one-off mutants, plant genomes appear to be rife with this form of diversity, so much so that “reference genomes” are now widely thought of as providing only a snapshot of the “pangenome” that actually characterizes a species (or group of species) (Golicz et al., 2016; Danilevicz et al., 2020). Different rice lines, for example, collectively contain at least 1.5 million SVs (Fuentes et al., 2019), and even in a relatively limited sampling of 19 maize inbreds and 14 teosintes, approximately 4000 *genes* experience either CNV or PAV (Swanson-Wagner et al., 2010). Similarly, about 9% of the 26,000 genes in a sampling of 80 *Arabidopsis* lines are missing in at least one line (Tan et al., 2012), and in a sampling of 115 cucumber lines, variation caused by SVs affects 1676 genes (Zhang et al., 2015). Accordingly, there is every reason to suspect that CNVs and PAVs can affect phenotypes and be relevant to natural selection and adaptation.

Evidence in support of this assertion now abounds, some from natural systems (Winzer et al., 2012; Flagel et al., 2014), but mostly from the crop literature (e.g., Wang et al., 2015; Zhang et al., 2015; Gabur et al., 2019; Schiessl et al., 2019). Importantly, from the standpoint of the present article, examples from polyploid crops are accumulating (Gaeta et al., 2007; Gabur et al., 2019; Schiessl et al., 2019), including oilseed rape (*Brassica napus*), potatoes (*Solanum tuberosum*), and bread wheat (*Triticum aestivum*). Traits for which SVs have been implicated as causative include some that are readily envisioned to be responsive to natural selection, such as flowering time and frost tolerance (Gabur et al., 2019; Schiessl et al., 2019). Given the scale and scope of SVs in all plants studied to date, it seems likely that their role in adaptive processes will increasingly be recognized as important, and especially in the adaptation and diversification of nascent allopolyploids, which are forged from the merger of two genomes that bring to the union differing suites of SVs.

In addition to small scale SVs affecting copy number or presence/absence of genes, exons, small repeats and the like, larger structural mutations abound in polyploids, resulting from processes such as reciprocal and non-reciprocal homoeologous exchanges (HEs) (Mason and Wendel, this issue). These mutations, which affect genomic regions ranging from smaller telomeric regions to interstitial segments to entire chromosome arms, have the capacity to simultaneously alter genic and non-genic PAVs and copy number dosages on a massive scale. Classically recognized as homoeologous translocations or transpositions, the genomics era and the successful sequencing of polyploid plants ushered in an increasing realization that HEs represent a fundamental mechanism of allopolyploid genome evolution and for generating diversity. Recent illustrative examples include peanuts (*Arachis hypogaea*; Bertioli et al., 2019; Zhuang et al., 2019), *Tragopogon* (Chester et al., 2012), quinoa (*Chenopodium quinoa*; Jarvis et al., 2017), *Brassica* (Hurgobin et al., 2018; Samans et al., 2018), tobacco (*Nicotiana tabacum*; Chen et al., 2018) and allopolyploid rice (constructed from *Oryza sativa subsp. indica* × *subsp. japonica*; Sun et al., 2017; Li C. et al., 2019). Increasing evidence suggests that in many systems HEs may occur genome-wide and be sequential, ongoing, and variable in size. In principle, this process can generate a limitless pool of genetically variable progeny over time, with each genomic combination carrying its own particular suite of chromosome segment copy numbers. One might imagine that the immense range of genomic diversity generated by an ongoing process of HE would be paralleled by phenotypic diversity, and thus it might represent a potent force for adaptation and evolutionary change in polyploids.

Hints that HEs might generate selectively relevant phenotypic diversity during allopolyploid evolution extend back at least to 2007 (Gaeta et al., 2007), and their association with PAVs has now been clearly established (Sun et al., 2017; Hurgobin et al., 2018; Li C. et al., 2019). In *Brassica*, HEs affect flowering time, disease resistance, and glucosinolate metabolism (Hurgobin et al., 2018). In rice allopolyploids, genome-wide gene expression and methylation states are massively altered by HEs, which also are associated with diverse phenotypes (Sun et al., 2017; Li C. et al., 2019; and unpubl.); most impressively, these outcomes arise in even the first few generations of selfing from a single founder following artificial allopolyploid synthesis (e.g., Chester et al., 2012; Sun et al., 2017; Hurgobin et al., 2018), demonstrating that HEs likely are a potent force for evolutionary novelty following allopolyploid speciation, at least in some groups.

### Whole-Genome Effects

A poorly understood but undoubtedly significant dimension of polyploidy concerns the evolutionary relevance of the myriad cascading effects set in motion by the doubling (in the case of autopolyploidy) or summing of genome sizes into a common nucleus. This additivity, by itself, is known to trigger diverse regulatory alterations in gene expression, translation, biosynthesis of metabolites and structures, cells sizes and shapes, organ size, physiology, and almost any aspect of plant development that one studies. Even at the level of the genome, recent studies using chromosome conformation capture, or Hi-C (Grob, 2020), have demonstrated that genome merger and WGD also dramatically alter the positional relationships and associations among chromosomes in the nucleus. A case in point is the remarkable study by Wang et al. (2018), who showed that in allopolyploid cotton, the suites of Topologically Associated Domains (TADs) within and between chromosomes are significantly altered by allopolyploidy relative to the diploid progenitors, and that some homoeologous chromosomal regions become spatially associated whereas others do not. Similarly, Zhang et al. (2019) recently showed that in autopolyploid *Arabidopsis thaliana*, chromosome doubling led to an increase in interchromosomal interactions and decreased association of more closely adjacent intrachromosomal sites. The effects of these types of spatial and organizational alterations on gene expression dynamics and all of the downstream reverberations that lead to phenotypes are largely unknown. Yet some data are beginning to close this circle; Zhang et al. (2019), for example, also showed that the altered chromatin interactions were associated with specific changes in gene expression and histone modifications that might affect phenotypes, including for the key flowering regulator *Flowering Locus C.*

Above the level of the genome, a burgeoning but highly fragmented literature exists bearing on one or more aspects of the suite of scaling and stoichiometric changes set in motion by polyploidy, as elegantly and comprehensively reviewed recently by Doyle and Coate (2019). Notwithstanding the examples cited in their review and in other parts of the present perspective piece, the fact remains that nearly all polyploidy-induced phenotypes, structural, metabolic, or physiological, that one might consider phenotypically relevant to adaptation, in either natural settings or in domesticated plants, represent emergent, downstream features of complex cascading networks of genic, regulatory and biosynthetic programs. As such, it is perhaps unsurprising that understanding the “genotype to phenotype” (G-to-P) mapping equation remains elusive for nearly all traits distinguishing diploid from allopolyploid congeners. Partial solutions to the G-to-P equation are provided for some complex traits using tools from multiple “omics” and scales. Examples including ploidy-related invasiveness potential in goldenrods (Wuet al., 2020), growth rate and phenotypic traits such as cell size and cell wall composition among *A. thaliana* plants having different levels of autopolyploidy (Corneillie et al., 2019), and even the rapid rise to global prominence of angiosperms following ancient WGD events (Simonin and Roddy, 2018). As noted by Doyle and Coate in citing Don Levin nearly 40 years ago (1983), “*the role of polyploidy per se in the development of evolutionary novelty remains one of the outstanding questions in flowering plant evolution*”. At the same time, Doyle and Coate offer promising ideas for a research agenda directed at this question, and as pointed out elsewhere in the present review, the tools and technologies available today are getting us closer to this holy grail. Some of these are alluded to in the following section.

## Responses at the Gene Expression and Regulatory Level

### Duplicate Gene Expression Evolution

One of the revelations of the genomics era, unsurprising in hindsight, is that the merger of two or more differentiated genomes, in the case of allopolyploidy, or the doubling of a single genome, in the case of autopolyploidy, causes massive, genome-wide alteration in gene expression patterns. These alterations accompany both hybridization and polyploidy separately, encompassing both immediate or short-term consequences of genome merger as well as evolved responses that arise over thousands to millions of years subsequent to WGD (Flagel et al., 2008). This temporal partitioning is useful when thinking about the evolutionary relevance of gene expression evolution, as it seems likely that many or perhaps most evolved responses were in fact enabled by relatively ancient polyploidy events, but that the adaptive signatures of such evolutionary change remain either obscure or are no longer evident. One example of this might be the well-known “radiation lag-time” hypothesis (Schranz et al., 2012; but see Tank et al., 2015; Landis et al., 2018), which was proposed to explain an observation of a delay, or lag phase, between inferred ancient polyploidy events and diversification. Irrespective of the effects of polyploidy on net diversification rates, the notion that doubled genomes may “have time” to generate adaptive phenotypes represents an important idea for understanding the evolutionary potential of polyploidy. Phrased alternatively, unlike the case in conventional diploids, the additive complement of genes, regulatory elements, and other genomic components that comprise a nascent polyploid (auto- or allo-) represent a vast storehouse of raw material for later, and perhaps much later adaptive responses to environmental change, niche expansion, or any other biotic or abiotic evolutionary opportunity.

Numerous phenomena and analytical frameworks are encompassed by the terms “expression evolution” or “regulatory evolution,” ranging from those focused on single pairs of duplicated genes (homoeologs) to others involving entire networks of coexpression for hundreds to thousands of duplicated genes. Many of these topics have been amply reviewed (Yoo et al., 2014; Soltis et al., 2015, 2016; Wendel, 2015; Panchy et al., 2016; Van de Peer et al., 2017; Bird et al., 2018; Cheng et al., 2018; Wendel et al., 2018). Here our attention is directed at the connections, known or suspected, between expression or regulatory evolution and plant phenotypes.

An early and illustrative example of differential homoeolog expression (“homoeolog bias”; Grover et al., 2012), is from allopolyploid (AD genome) cotton (*Gossypium*), where it was shown that the two co-resident, alternative homoeologs (A, D) had differential contributions to the total transcript pool, and that this homoeolog ratio varied widely among genes (Adams et al., 2003). In the most extreme cases, reciprocal silencing of alternative homoeologs was observed for different tissues; for example, in petals and stamens, only the D homoeolog was expressed, whereas in ovary walls of the same flower, only the A homoeolog was expressed. Whereas not all genes in every allopolyploid exhibits this degree of homoeolog bias, the phenomenon itself is an ubiquitous feature of polyploids (Grover et al., 2012; Yoo et al., 2014), and it may be that there are few if any homoeologous gene pairs in any species that contribute equally to the transcript pool in all tissues. Thus, homoeolog bias appears to be a rule rather than an exception. Yet differing transcript ratios may not be evolutionarily meaningful in terms of protein function if the genes encoded by the two homoeologs are functionally equivalent and do not differ in expression domains. Accordingly, evolutionary relevance is more likely in situations where different “function” has been inferred for two homoeologs, either prior to hybridization and WGD or as an evolved feature subsequent to polyploidization. These two cases are quite different in terms of our understanding of the timing of evolutionary divergence, that is, whether homoeolog functional differences arose at the diploid or the polyploid level. Distinguishing between these two cases often is not possible because the progenitor diploids are unknown or extinct, and in this respect a number of plant genera have become particularly useful models (Soltis et al., 2016), including *Gossypium, Nicotiana, Arabidopsis, Tragopogon, Senecio, Glycine, Brassica, Spartina, Aegilops-Triticum*.

Functional divergence and other forms of neofunctionalization of homoeologs involves the acquisition of novel expression domains, interactions, or protein function following their merger in a common polyploid nucleus, and it is the duplicate gene outcome of most interest to adaptation. Other possible fates of duplicated genes, not wholly separable from neofunctionalization, include subfunctionalization, where ancestral aggregate expression space or function is partitioned developmentally or in a tissue-specific fashion following polyploidy, non-functionalization (mutational loss of one or the other homoeolog), dosage subfunctionalization (Gout and Lynch, 2015), and several other related outcomes. These and other possibilities regarding the evolutionary fates of gene duplication have been extensively reviewed (e.g., Conant and Wolfe, 2008; Flagel and Wendel, 2009; Panchy et al., 2016; Cheng et al., 2018). While these categorizations are conceptually useful, most empirical examples defy a simple characterization, as divergence often involves multiple steps and various combinations of duplication, loss, subfunctionalization and neofunctionalization.

An excellent example of neo- and subfunctionalization concerns a pair of genes in the Brassicaceae duplicated by a polyploidy event about 23 MYA (Liu and Adams, 2010). The two paralogs SHORT SUSPENSOR (SSP), involved in paternal control of zygote elongation in *A. thaliana*, and Brassinosteroid Kinase 1 (BSK1), involved in brassinosteroid signal transduction, have diverged in function, with BSK1 retaining its ancestral role in hormone signaling. SSP, however, diverged following duplication to acquire a role in zygote elongation, while losing its plesiomorphic role in signal transduction through loss of its kinase domain. A second illustrative example concerns the fate of duplicated MADS-box transcription factors in columbine (*Aquilegia*) flowers (Sharma and Kramer, 2013). Duplication of ancestral APETALA3 genes in the Ranunculaceae appears to have been followed by a combination of neo- and subfunctionalization of different paralogs, while contributing to the evolution of a novel floral whorl where the innermost stamens have been converted to sterile staminodes. A third example concerns the free-threshing Q locus in hexaploid wheat (Zhang et al., 2011), which encodes an AP2-type transcription factor. This locus has a complex history of duplication and differential paralog loss among diploid wheat lineages during the evolution of *Triticum/Aegilops*. Diverged paralogs were reunited in a common nucleus with polyploid wheat formation, and this was followed both by protein evolution in one homoeolog and pseudogenization/subfunctionalization of other homoeologs. This example entails a complex combination of ancient paralogy, non-functionalization, reunion of divergent paralogs, and interaction of subfunctionalized homoeologs. These specific examples of post-duplication evolutionary divergence differ greatly in their timing, underlying molecular bases, and ecological settings. It remains to be seen whether commonalities in any of these attributes will emerge in the future as additional examples are revealed.

In addition to examples involving duplicated genes connected to specific phenotypes, broader surveys of patterns of gene retention following polyploidy have implicated certain classes of genes in morphological innovation or in adaptation to various environmental conditions. Transcription factors, for example, have been shown to be preferentially retained following ancient polyploidy events (De Smet et al., 2013; Li et al., 2016), and many of these retained ancient homoeologs later become modern paralogs with differing roles in regulatory development and plant morphology (reviewed in Rensing, 2014). Schilling et al. (2020) studied 201 wheat MIKC-type MADS-box genes, showing both preferential retention and even gene family expansion for genes involved in adaptation to different environmental conditions, abiotic and biotic stresses, and flowering time. These and other studies indicate that transcription factor retention and divergence appears to be an important aspect of polyploid diversification. Insight into the nature of this preferential retention was recently provided by Panchy et al. (2019), who showed that rapid evolution of *cis* binding sites generates novel TF expression patterns that lead to subfunctionalization and neofunctionalization as well as complex combinations of new and ancestral expression states.

Additional insights into the evolution of duplicated genes has emerged from studies of suites of genes tracing to a common progenitor genome, rather than a common class or category of gene. An exemplar study in this respect is that of De Smet et al. (2017) who demonstrated a pattern of coordinated gene expression following ancient polyploidy in *A. thaliana*. In this case a suite of 92 homoeologous gene pairs were identified which shared a common pattern of tissue-specific gene expression, but with the two homoeologous suites being partitioned such that one was expressed mostly in aerial tissues while the other was expressed predominantly in roots. This remarkable example of coordinated homoeolog evolution among diverse sets of genes has parallels to recent discoveries from using coexpression network approaches, discussed below.

### Altered Epigenetic Landscapes

The observation that global patterns of gene expression are dramatically altered by a change in ploidy (above) led to the supposition that at least part of this response must have an epigenetic basis. This supposition was confirmed by studies of synthetic polyploids and interspecific F1 hybrids, plants in which there has been virtually no chance for mutational effects yet which exhibit massive gene expression modification (Adams et al., 2003, 2004; Flagel et al., 2008; Yoo et al., 2014; Song and Chen, 2015; Ding and Chen, 2018). This regulatory rewiring of the transcriptome likely has numerous combinatorial causes stemming from altered cell and nuclear volumes (Doyle and Coate, 2019), biochemical and biophysical stoichiometric disruptions (Bottani et al., 2018; Hu and Wendel, 2019), and changed *cis* and *trans* controls on gene expression (Bao et al., 2019). All of these phenomena are related to various forms of epigenetic modification and chromatin remodeling, including changes in DNA methylation and various histone modifications, for both autopolyploids and allopolyploids (Song and Chen, 2015; Ding and Chen, 2018).

A beautiful example of the relevance of these epigenetic phenomena to novel plant phenotypes associated with allopolyploidy concerns flowering time in domesticated forms of allotetraploid (AD genome) cotton (Song et al., 2017). Wild forms of both *Gossypium barbadense* and *G. hirsutum* contain hypermethylated forms of both homoeologs (A, D) of CONSTANS-LIKE 2 (COL2) and are photoperiod sensitive. As a consequence of domestication, however, DNA methylation was lost for the D homoeolog, leading to higher expression of COL2D and the all-important photoperiod insensitivity that allowed cotton production to thrive outside of the subtropics. A second example concerns circadian clock genes in *Arabidopsis* hybrids and allotetraploids (Ni et al., 2009), where histone modifications were linked to increased biomass, vigor, and starch accumulation. A third example of an epigenetically mediated plant trait accompanying polyploidy, recent or ancient, is the possible epigenetic neofunctionalization of a parentally imprinted polycomb group protein in grasses (Dickinson et al., 2012), which may have contributed to the evolution of the globally important large endosperm found in cereals. Finally, Lu et al. (2020) recently observed that the polyploid populations of *Solidago canadensis*, which have a more southerly distribution than their diploid counterparts, and which have a lower freezing tolerance, had lower expression levels but more copies of a key gene (ScICE1) involved in freezing tolerance. In this case the authors suggested that promoter DNA methylation has repressed expression, leading to polyploid adaptation accompanying range expansion.

It seems likely that we have only just begun to understand the relationships among epigenetic responses to polyploidy and novel phenotypes or adaptation, but given the scale and the scope of both polyploidy and its unavoidable epigenetic consequences, it seems probable that many new examples will soon emerge.

Finally, we note the potentially important observation that reciprocal homoeolog silencing, as described for different organs of the same plant in *Gossypium* (Adams et al., 2003, 2004), can arise immediately upon polyploid formation. Moreover, the same tissue-specific expression pattern has been observed in natural allopolyploids 1–2 million years following initial polyploid formation. This suggests the tantalizing possibility that there has been stable maintenance of an epigenetic mutation since the initial formation of allopolyploid *Gossypium*. Adams et al. (2004) noted the important temporal dimension of this epigenetic suppression of gene expression, suggesting that epigenetic subfunctionalization may provide a selective constraint favoring duplicate gene retention, in that both homoeologs would be necessary to enable expression in alternative tissues. To the extent that this is true, epigenetic subfunctionalization might provide a latent reservoir of hundreds to thousands of genes, which “become exposed to an evolutionary filter only after additional epigenetic and genetic evolution” (Adams et al., 2004, p. 2225) perhaps thousands to millions of years later. The scale of the phenomenon of homoeolog bias and reciprocal silencing, as noted above, suggests that epigenetic maintenance via subfunctionalization may prove to be a significant facet of polyploid evolution.

### Small RNA Duplication and Divergence

Another genomic facet of hybridization and polyploidy is the attendant combining of diverged populations of small RNAs (sRNAs), including microRNAs (miRNAs) and several classes of small interfering RNAs (siRNAs) (Ng et al., 2012; Wendel et al., 2016; D’Ario et al., 2017). Each of these classes of 21 to 24 nucleotide RNAs has a suppressive effect on either gene or transposable element (TE) expression, and all are subject to the novel *trans*-regulatory controls established by genome merger. As such, the combination of two populations of diverged sRNAs has the potential to change patterns of gene expression, TE activity, and all of the developmental programs and phenotypes that might result from this altered regulatory environment. Micro RNAs, for example, are known to be important players in stress responses and other forms of ecological adaptation (Song et al., 2019; Yu et al., 2019), which raises the possibility that these small regulatory molecules might facilitate neofunctionalization of duplicated stress-related and other signaling genes (Palacios et al., 2019).

A growing literature attests to the effects of polyploidy on expression of small RNAs (reviewed in Ng et al., 2012; Wendel et al., 2016). As with protein-coding genes, in most studies at least some sRNAs are non-additively expressed (e.g., Li et al., 2014; Xie and Zhang, 2015; Cavé-Radet et al., 2019; Palacios et al., 2019; Wei et al., 2019), being subject to novel *trans* regulation. sRNA non-additivity in polyploids may be unsurprising in that this also has been observed within species as well, as in maize hybrids (Crisp et al., 2020). For miRNAs, this non-additivity will affect downstream gene regulation, so in principle non-additivity should have consequences for plant phenotypes; for siRNAs, non-additive siRNA expression could, in *trans*, affect epigenetic silencing or activation of TEs (transposon elements), which could also exert effects on neighboring protein-coding genes and eventually cause phenotypic alterations (see below). There are, however, surprisingly few examples of where sRNA divergence has been causally connected to novel traits in polyploids. In cotton allopolyploids, for example, homoeologous MYB2 transcription factors, regulated by miR828 and miR858, are important regulators of cotton fiber development (Guan et al., 2014). These miRNAs have functionally diverged with respect to their targeting preferences, and are inferred to contribute in a novel manner to polyploid cotton fiber growth. It seems probable that additional examples will soon emerge from functional analysis of the omnipresent alterations in sRNA expression in polyploids.

### Altered *Cis* and *Trans* Relationships

Allopolyploidy entails the merger of two suites of partially diverged *cis-* and *trans*-regulatory elements, and as such gene expression is expected to be altered due to the several new forms of regulatory interactions in the polyploid nucleus (Bottani et al., 2018; Hu and Wendel, 2019). These expectations recently were illuminated in an experiment where reciprocal F1 hybrids were constructed between cultivated and wild accessions of the allotetraploid cotton species *G. hirsutum* (Bao et al., 2019). Although the goal was to understand the nature of the domestication process, the study revealed a surprisingly high level of *trans*-regulatory control of gene expression (54–64%), higher than observed in comparable studies in diploids. Bao et al. (2019) proposed the explanation that with the onset of allopolyploidy, *trans* factors throughout the genome instantaneously acquire duplicated homoeologous suites of *cis* elements with which to interact. This aspect of allopolyploidy generates extensive and novel *cis-trans* interactions, especially for *trans* variants. As noted by Bao et al. (2019), “This phenomenon of enhanced *trans* regulatory evolution may be a general and previously unrecognized feature of polyploidy, perhaps helping to explain evolutionary novelty in recently formed allopolyploid plants.” It seems probable that in the next few years specific examples will emerge where novel phenotypes are causally connected to these new regulatory interactions.

## Combinatorial Genomic and Expression-Level Responses

### Novel Cytonuclear Combinations

Allopolyploid formation not only results in the merger of two nuclear genomes, but because it is directional there is a pollen donor and an ovule donor. The latter is the source of the cytoplasm with its mitochondria and plastids in perhaps 80% of angiosperms (Corriveau and Coleman, 1988), and accordingly the nascent allopolyploid and all descendant lineages have organellar genomes tracing to the maternal diploid parent. This directional asymmetry also is accompanied by a genic imbalance, as polyploidization results in a doubling of nuclear gene content (for a tetraploid) with a more uncertain quantitative effect on cytoplasmic genome number (Sharbrough et al., 2017; Doyle and Coate, 2019). Because organellar genomes diverge during diploid divergence, in many of the same ways as those discussed above for nuclear genomes, and because many aspects of plant physiology and development involve finely tuned integration of plastidial and mitochondrial processes with anterograde and retrograde nuclear-organellar signaling, there has been a long interest in the evolutionary dimension of this cytonuclear relationship (reviewed in, e.g., Bock et al., 2014; Sharbrough et al., 2017; Fishman and Sweigart, 2018). This work includes observational, experimental, and statistical evidence bearing on the fitness of different cytonuclear combinations within and between species (Bock et al., 2014; Case et al., 2016; Roux F. et al., 2016) as well as a vast literature on cytonuclear incompatibility (Fishman and Sweigart, 2018) and the important topic of cytoplasmic male sterility in crop plants (Chen et al., 2017). That specific cytonuclear combinations can affect plant phenotype has now been firmly established. This was recently convincingly demonstrated by Flood et al. (2020) for 1,859 phenotypes in all possible cybrid combinations among seven *A. thaliana* lines.

With respect to hybridization and polyploidy, mounting evidence suggests that cytonuclear molecular coevolutionary responses will be common. One source of evidence is “global”; for example, nuclear genes encoding organellar processes are preferentially restored to single-copy status following polyploidy events (De Smet et al., 2013), presumably to restore “normal” stoichiometric relationships with organellar-encoded proteins. Another example is that of the D-genome wheat species *Aegilops tauschii*, which has a homoploid hybrid origin and which has preferentially retained genes from its maternal A-genome ancestry for nuclear genes that encode cytonuclear enzyme complexes (Li N. et al., 2019). At a more granular level, Gong et al. (2012, 2014) studied RuBisCO in allopolyploids of five different model genera, *Arabidopsis, Arachis, Brassica, Gossypium*, and *Nicotiana*, demonstrating that paternal copies of the nuclear-encoded small subunit (*rbcS*) experience gene conversion such that their sequences became maternal-like, and also that there was preferential expression of the maternal *rbcS* copies. These putative coevolutionary responses were not found, however, in some other genera, e.g., *Tragopogon* (Sehrish et al., 2015) and *Cucumis* (Zhai et al., 2019).

A potential connection to phenotype, and an example that integrates several of the mechanisms discussed in the present review, is from Wu et al. (unpublished), who studied synthetic allopolyploids synthesized from reciprocal crosses between rice *O. sativa* subsp. *sativa* and subsp. *japonica.* Each generation of selfing was accompanied by multiple homoeologous exchanges (see earlier discussion), collectively affecting all members of the chromosome complement, such that after four generations of selfing the resulting individuals were genomic mosaics of the two founding parents. Importantly, some genomic regions were preferentially and reciprocally biased with respect to maternal vs. paternal progenitor in that they were repeatedly associated with only one of the two parental cytoplasms, whereas other chromosomal regions were exclusively maintained as heterozygotes, suggesting hetero-cytonuclear interactions.

As with many of the other phenomena discussed here, the foregoing synopsis suggests an adaptive dimension to cytonuclear interactions that may be set in motion through hybridization and genome doubling.

### Effects on Transposable Element Activity

Transposon elements are mobile genetic elements that account for a large but variable fraction of virtually all eukaryotic genomes, including plants. As an example, LTR-retrotransposons, which together with miniature inverted-repeat transposable elements (MITEs) constitute the most prevalent and active class of TEs in plants (Casacuberta and Santiago, 2003), account for only 2.5% of the small and compact genome of *Utricularia gibba* (Ibarra-Laclette et al., 2013) but 90% of the gigantic genome of *Fritillaria* spp. (Ambrožová et al., 2011). In fact, TE proliferation and TE elimination by transposon-mediated recombination and deletion-biased double strand break (DSB) repair are two of the primary drivers of genome expansion and shrinkage during land plant evolution (Pellicer et al., 2018). These processes can play out saltationally to generate large changes in genome size even over short evolutionary time scales. As an example, the proliferation of a few retrotransposon families explains the doubling of the genome size of *Oryza australiensis* relative to rice (Piegu et al., 2006). Similarly, the 3-fold difference in genome sizes among different diploid cotton (*Gossypium*) species reflects the differential dynamics of TE proliferation and clearance since these species shared a common ancestor (Hawkins et al., 2009). Even within species there can be extensive TE polymorphism (Carpentier et al., 2019; Noshay et al., 2019). Thus, the TE component of plant genomes is highly variable in plants, even among closely related species and often within species.

In the context of the present review, the merger of two different genomes will inevitably combine two different TE populations, which is of particular relevance because the presence and mobility of TEs impact genomes in many ways. First, their transposition induces new insertions, which in most cases will be selectively neutral or slightly deleterious, but in other cases could provide a selective advantage (Arkhipova, 2018). Second, their repetitive nature offers numerous pairs of sequences that can recombine, and accordingly, TEs are a major source of structural variants, including genic CNV and PAV, as discussed earlier (see ***Structural diversity***, above, see also Fuentes et al., 2019). And third, TEs are a rich source of new genes and gene functions and can directly or indirectly regulate gene expression (Lisch, 2013). Indeed, TEs are an important source of promoters and transcriptional regulatory elements. Transcription is the first step of transposition, and TEs contain internal promoters to facilitate their own expression. The insertion of TEs within or close to genes can therefore alter the expression of neighboring genes by providing additional transcription factor binding sites or alternative promoters and splicing signals, a phenomenon frequently found in both animal (Chuong et al., 2017) and plant genomes (Lisch, 2013).

Transposon elements accumulate in certain regions of the genome (e.g., centromeres and pericentromeric regions) where they can fulfill important structural functions, as for example supporting the specification and function of centromeres (Lermontova et al., 2015). But this non-homogeneous accumulation in chromosomes also impacts genic evolution. In filamentous fungi, the “two-speed genomes” concept has been developed to describe the concentration of the fast-evolving virulence effectors in TE-rich compartments of the genome (Dong et al., 2015). The formation of TE islands and differentiated genomic regions showing distinct evolutionary rates could allow for the rapid evolution required for adaptation to new environments while preserving the basic genic machinery (Schrader and Schmitz, 2019), as has been shown for the invasive ant *Cardiocondyla obscurior* (Schrader et al., 2014). Although this phenomenon has not been described as such in plants (Lanciano and Mirouze, 2018), there are accumulating examples of TE impact on the evolution of different plant resistances to biotic and abiotic stress, such as disease resistance in pepper (Kim et al., 2017) and aluminum resistance in a wide range of land plants (Pereira and Ryan, 2019), and it has long been known that plant disease resistant genes frequently concentrate in resistance gene clusters which are also rich in TEs (Richter and Ronald, 2000). Moreover, the differential distribution of genes with respect to their age or function in genomic compartments defined by a different TE content has recently been shown in plants, including tomato (Jouffroy et al., 2016) and melon (Morata et al., 2018b), suggesting that TE-rich compartments may facilitate rapid adaptation in plants. In addition, TEs can also modify the local recombination rate along chromosomes. Indeed, a recent study in natural populations of *Arabis alpina* has shown that TEs can create linkage disequilibrium blocks defining adaptive loci that concentrate environment-responsive genes (Choudhury et al., 2019).

Given their ubiquity, extraordinary variability even within species, and propensity to function as genomic architects, it is likely that TEs are important internal drivers of plant evolution and adaptation. However, TEs also pose a hazard for genome integrity, as they are a cause of potentially deleterious mutations and chromosome instability. For this reason, TE activity is highly repressed by different mechanisms, the most important being epigenetic silencing driven by DNA methylation (Kim and Zilberman, 2014; Zhang H. et al., 2018). TEs are the main target of epigenetic silencing marks in the genome, and TE distribution closely matches that of DNA methylation (El Baidouri et al., 2015). The equilibrium between efficient silencing mechanisms to control TEs, and the escape of some TEs from this control under particular circumstances, allows for TE maintenance and genome plasticity while maintaining genome integrity. TEs are mostly quiescent and are only activated in particular developmental stages (Martínez and Slotkin, 2012) or under stress (Galindo-González et al., 2017). This activation of TEs by stress, and probably also during development, likely is the result of the combination of the presence of specific activator sequences in TE promoters (Galindo-González et al., 2017), and the alleviation of epigenetic silencing in these situations (Gutzat and Mittelsten Scheid, 2012). The activation of TEs under stress allows for the generation of new variability in situations to which the genome is not necessarily well-adapted. In addition, the insertion of TEs with stress-related promoters close to genes could result in the stress-related expression of a new set of genes offering new possibilities for adaptation to new environmental conditions. Interestingly, TE integrations are frequently not random, and it recently has been shown that some LTR-retrotransposons preferentially target environmentally responsive genes (Quadrana et al., 2019), generating new genetic or epigenetic variability that could facilitate rapid adaptation to new environments.

Although LTR-retrotransposons are the most obvious candidates for altering adjacent gene expression, other TEs also have this potential. In particular, MITEs, which are present in high copy number and are enriched in genic regions (Casacuberta and Santiago, 2003), frequently contain transcription factor binding sites (Morata et al., 2018a). In addition, TEs of different types have been shown to alter gene splicing upon insertion. As an example, an Helitron insertion into a host susceptibility factor gene causes its alternative splicing leading to resistance to maize rough dwarf disease (Liu et al., 2020). Moreover, as noted above, TEs are the main target of epigenetic silencing, and therefore, the insertion of a TE within or close to a gene may bring the silencing machinery to this gene altering its expression (Zhang H. et al., 2018). In addition to bringing new promoters and promoter elements, TEs can also be the source of small RNAs that regulate gene expression (McCue and Slotkin, 2012), and in particular that of defense-related genes (Poretti et al., 2020), and it has been recently shown that they can also contribute long non-coding RNAs that regulate plant stress responses (Lv et al., 2019). TEs are therefore an important force that facilitates plant, and also animal (Rech et al., 2019) and yeast (Esnault et al., 2019), adaptation to stress. This confirms McClintock’s revolutionary hypothesis on the role of mobile genetic elements in overcoming the threat of environmental shock by reorganizing the genome (McClintock, 1984).

McClintock’s proposal regarding the “shock” that follows genome merger has been amply evidenced by data showing that plant hybridization and polyploidization frequently trigger TE activation (Vicient and Casacuberta, 2017). Transcriptional activation of TEs has been reported in synthetic *Arabidopsis* polyploids (Madlung et al., 2005), wheat amphiploids (Kashkush et al., 2003), allopolyploid coffee (Lopes et al., 2013), and in rice lines derived from introgressive hybridization with *Zizania latifolia* (Wang et al., 2010), among others. This activation of TEs in hybrids and polyploids has been shown to be accompanied in many cases by a modification of the siRNAs that target TEs (Springer et al., 2016). Moreover, TE mobilization and increase in copy number has also been reported for different hybrids and polyploids, including tobacco (Petit et al., 2010), wheat (Yaakov and Kashkush, 2012) and *Brassica* (Sarilar et al., 2013) allopolyploids, in *Biscutella laevigata* autopolyploids (Bardil et al., 2015), and in sunflower hybrids (Kawakami et al., 2010). However, in other allopolyploids or interspecific crosses no evidence of an increase of TE content was shown. For example, no changes were observed in TE regulation after an interspecific cross between *A. thaliana* and *A. lyrata* (Göbel et al., 2018), and no TE burst was detected after polyploidization of *A. arenosa* (Baduel et al., 2019). Moreover, an increase of TE-related siRNAs was recently reported in *Spartina* allopolyploids, suggesting a strengthening of TE repression accompanying polyploidization (Cavé-Radet et al., 2019).

An important dimension of TE mobilization following genome merger is that this varies among TE families, and the same TE may proliferate in some polyploids while being eliminated in others. As an example, *Sabine* retrotransposons proliferated following polyploidy in some *Aegilops* (wheat) polyploids while being eliminated in others (Senerchia et al., 2014), even while other retrotransposon families (*BARE1*, *Romani*) experienced a more uniform proliferation. Different TE families are regulated dissimilarly and their responses to the methylation and siRNA changes that accompany genome merger may vary accordingly. At present there is little understanding of this extraordinary variation in TE responses to polyploidy, but we can imagine that this reflects an equivalent level of diversity with respect to divergence in progenitor TE populations and their repression and activation dynamics among parental diploids in each genus. In this respect, it has recently been shown that there is a correlation between TE mobilization in *Nicotiana* allopolyploids and the quantitative imbalance in parental TE loads (Mhiri et al., 2019). But even in the absence of TE activation, polyploidy may result in an increase in TE content due to relaxed purifying selection at duplicated loci (Ågren et al., 2016; Baduel et al., 2019).

In summary, merging two different genomes, with or without polyploidization, will combine in a single genome two different TE populations, together with the siRNAs that target them, and this may result in changes in the epigenetic modifications at TEs and neighboring genes and in the regulation of genes and TEs.

Given the prevalence of TEs, their frequent association with genes, and their potential for insertional mutagenesis following genome merger, it seems probable that they are important players in the creation of novel traits and adaptation in polyploids. As explained above, the importance of TEs in creating phenotypic variability in plants is well established (Lisch, 2013), and examples of the role of TEs in creating new adaptive alleles are slowly accumulating. For example, TEs have been recently shown to create adaptive alleles that modify flowering time (Huang et al., 2017; Liang et al., 2019; Niu et al., 2019), facilitate local climate adaptation (He et al., 2018), and trigger new responses to biotic (Poretti et al., 2020) and abiotic (Lv et al., 2019) stresses, sometimes by facilitating the formation of complex biosynthetic pathways (Xu et al., 2017). However, direct evidence of TEs generating new adaptive phenotypes as a consequence of the merging of two genomes remains scarce. As one example, for TE families targeting environmentally responsive genes, activation may introduce target variability at these loci, potentially facilitating rapid adaptation (Quadrana et al., 2019). An added dimension to this scenario is that relaxed purifying selection in polyploids (due to duplication) can also result in accumulation of TEs close to environmentally responsive genes (Baduel et al., 2019). The combination of TE activation and the relaxed purifying selection that frequently accompanies genome merger provides a powerful mechanism for the generation of novel allelic combinations for stress-related or other adaptive responses. We note that in principle adaptation may be facilitated by selection on even a single TE insertion affecting a single gene, as illustrated by the beautiful example of the peppered moth industrial melanism mutation (Hof et al., 2016), or it might involve the evolution of complex pathways, as has been shown for the nicotine synthesis in *Nicotiana* (Xu et al., 2017).

### Disrupted/Altered Regulatory Networks

It may be useful to consider that each of the many mechanisms introduced in the foregoing sections, from simple genic or regulatory SNPs through novel transposable element activity, ultimately shape phenotypes via their propagation through complex networks of gene regulation, transcription and translation, and higher order biochemical, physiological and biosynthetic processes (Gottlieb, 1984). To this extent, nearly all selectively relevant phenotypes likely represent emergent properties resulting from high-level multidimensional, interconnected meshworks of lower level “omic” processes. We expect that we are entering a period during which this omics-enabled view of adaptation and evolutionary change will rapidly expand, concomitant with the application of a suite of enabling technologies to model experimental and natural systems.

A foreshadowing of this form of evolutionary exploration is offered by the development of coexpression network analysis, in which entire transcriptomes are interrogated for patterns of genic and “modular” coexpression, or lack thereof. The logic for this rests on the assumption stated above, i.e., that genes work in concert rather than in isolation to generate phenotypes. Many aspects of genic coexpression network analysis have been amply reviewed (e.g., Serin et al., 2016; Emamjomeh et al., 2017; Ruprecht et al., 2017b; Contreras-Lopez et al., 2018; Joehanes, 2018; Rao and Dixon, 2019); here our attention is focused on applications involving adaptation following genome duplication and/or merger.

Recent studies have demonstrated that gene regulatory rewiring often follows duplication (Gupta and Tsiantis, 2018), and that entire modules of genic coexpression may be duplicated and retained in plants. Ruprecht et al. (2016), for example, showed that in *Arabidopsis* a module for cell wall biosynthesis has become replicated and deployed differentially for different types of cell walls. Pfeifer et al. (2014) showed that genic coexpression in bread wheat grains was partitioned into 25 “modules”, 23 of which contained biased suites of homoeologs from each of the hexaploid’s three co-resident (A, B, D) genomes. More recently, Takahagi et al. (2018) conducted coexpression analysis of 727 RNAseq data sets from bread wheat, reiterating and extending these findings of differential homoeolog composition of key modules (here meaning suites of coexpressed genes) involved in many biological processes, including chloroplast biogenesis, RNA metabolism, putative defense response, putative posttranscriptional modification, and lipid metabolism. In contrast, Alabdullah et al. (2019) examined how polyploidization in wheat affected meiotic genes, using 130 RNA-seq samples to define co-expressed gene modules. Among the three modules significantly correlated with meiosis, most genes retained all three homoeologous copies, and genes within these modules also exhibited balanced homoeologous expression. Though the foregoing studies were not designed to evaluate whether homoeologous-genome modular portioning of genic coxpression arose prior to or subsequent to polyploidization, it is likely that these modular structures represent network level transcriptomic adjustment to the polyploid condition.

Explicit tests of this diploid-polyploid temporal partitioning have recently been performed using allopolyploid (AD-genome) cotton (*Gossypium*), which contains two genomes (A, D) which diverged 5–10 million years ago (mya) in diploid lineages that became reunited during polyploid formation 1–2 mya. Hu et al. (2016) studied gene coexpression networks in developing seeds of diploid as well as allopolyploid species, Network comparisons among species indicated that the global network topology of allopolyploid cotton was asymmetric, resembling one of its two diploid progenitors (the A-genome diploid) more than that of the other (D-genome parent). A novel feature of this study was that it extended, by example, concepts of homoeolog bias, dominance, and transgressive expression to the network modular level. They further showed that the transcriptomic architecture in developing polyploid cotton seeds is a partial combination of the modules observed in the two diploid progenitors, and that domestication of wild allopolyploid cotton led to a more tightly integrated (more highly coexpressed) modular structure. These results for cotton seed development were recently extended to fiber development (Gallagher et al., 2020). Key results include the fact that notwithstanding a general preservation of network modular structure among the A- and D-genome diploids and the allopolyploid, fewer than a quarter of all homoeologs co-occured in the same module, showing substantial homoeologous expression rewiring (alteration of coexpression relationships) at the intramodular level. In addition, most modules exhibit D-homoeolog expression bias, with few showing A-homoeolog bias.

The preceding examples are illustrative of the various types of expression change that accompany polyploid formation, showing that not only is gene expression itself massively altered, but that this gene-level view has multiple parallels at the modular level of gene coexpression relationships. Yet, connections between these phenomena and demonstrations of adaptation or diversification remain mostly obscure, however, notwithstanding our growing understanding the relationships between modular genic content and biological processes. In this respect one promising approach is to combine coexpression network analysis with standard tools from population genetics, as exemplified in a marvelous recent study on *Theobroma cacao* from Brazil (Hämälä et al., 2020). Starting with the initial suggestion that evolutionary change often arises from allele frequency shifts simultaneously at multiple genes, Hämälä et al. (2020) studied genic coexpression relationships for 31 individuals from four geographically allopatric populations. Genes from modules enriched for specific biological processes were combined to explore whether they exhibited possible differential selection between populations, using a coexpression-module-based form of the widely used FST and dXY. They showed that modules associated with biological processes such as protein modification, flowering, and water transport were implicated in polygenic adaptation, “even though individual genes that are members of those groups do not bear strong signatures of selection.” Noting that this example is for possible differential adaptation of populations of a single diploid species rather than for the effects of introgression or polyploidization, it conceptually helps point the way to identifying cases of adaptation stemming from the latter speciation and diversification processes.

## Concluding Remarks

Decades of inquiry have generated much insight into the evolution of merged genomes following polyploidy and hybridization. For instance, we know that duplicated genes experience a diversity of evolutionary trajectories, being either lost, epigenetically silenced, or retained but with altered expression regulation and possible neofunctionalization or subfunctionalization, and that some of these outcomes may be tissue or organ-specific. For these and other consequences of genome merger discussed here, some have considered whether there might be evolutionary “rules” or mechanisms that are predictive of specific outcomes (Adams and Wendel, 2005; Doyle et al., 2008; Soltis et al., 2016; Wendel et al., 2018). One generality is that the early stages of genome merger and doubling profoundly impact the molecular, genomic and physiological machinery, as Barbara McClintock famously anticipated in her 1983 Nobel lecture, and as Feldman et al. (2012) captured in partitioning evolutionary change into “revolutionary,” that is, arising shortly after genome merger, versus “evolutionary,” namely change that accrues more gradually over time. Many empirical examples of phenotypic and genomic innovation have been discussed here, reflecting a broad spectrum of underlying mechanisms and phenotypes. Yet, notwithstanding the extraordinary advances of the last several decades and the increasing use of breathtaking technologies for probing genomes and transcriptomes, we still have only a rudimentary understanding of how genome merger generates phenotypic diversity and thereby contributes to evolutionary diversification. Also poorly understood is the relative importance of the many genomic responses discussed here to adaptive evolution or phenotypic innovation at the diploid vs. polyploid level. Both organismal processes entail genetic merger, which variously sets in motion a plethora of “omics” changes, as illustrated in Figure 1, but it is unknown which if any of these responses are more characteristic of diploid vs. polyploid evolution. Some progress in this direction has emerged from studies designed to assess this temporally partitioning, across multiple genera (e.g., Flagel et al., 2008; Xu et al., 2014; Edger et al., 2017; Zhang D. et al., 2018), but this clearly is an area that warrants further investigation.

It is of interest to consider the constraints that hinder our understanding of the genetic basis of phenotypic innovation that follows polyploidy. To be sure, we do not yet understand the full dimensionality of the genotype-to-phenotype equation, and thus our insight into phenotypes is limited by our present understanding of the propagation of information from the genome through all of the “omics” into something emergent that we call the phenotype (Casacuberta et al., 2016). In addition, though, we suggest that the early responses to genome merger and/or doubling represent only the tip of the iceberg compared to later evolutionary innovation, which ultimately was enabled or set in motion by genome merger but which remains latent, perhaps for millions of years, until ecological opportunity dovetails with novel genomic/omic recombinants. This temporal dimension is key to our perspective; genome merger sets the stage for both immediate and long-term evolutionary innovation. The retention of many to most duplicated genes and other genomic components in a polyploid serves as a massive reservoir of variation that may remain evolutionarily latent, perhaps for hundreds to thousands to millions of years. Only later, perhaps when exposed to altered selection pressures in novel environment, will this variation lead to phenotypic innovation, adaptation, and speciation. Yet this novel diversity may not have been possible without the ancient genomic infusion (or infusions) from interspecific gene flow. That is, long-term retention of duplicated genes, regulatory elements, and other genomic components, may be responsible for evolutionary diversification, even after extinction of the parental lineages and in novel environments relative to the progenitors.

This possibility is consistent with the so-called radiation lag-time model following WGD, formulated on the basis of phylogenetic and divergence-time data (Schranz et al., 2012). According to this hypothesis, successful diversifications in groups that experience ancient polyploidy do not arise due to the sudden genesis of novel key traits, but instead reflects subsequent phenomena over evolutionary time, including changing environmental conditions. This perspective is also in line with classical views that genome doubling provides a massive reservoir of duplicated genes for longer-term evolution of new functions (Stebbins, 1950; Ohno, 1970). Finally, as we note above, the possibility of epigenetic “subfunctionalization” offers a mechanism for selective retention of duplicate genes for later release from suppression and evaluation by natural selection (Adams et al., 2003, 2004).

Thus, there is now a confluence among classical notions and modern genomic perspectives regarding the importance of long-term persistence of latent variation generated by hybridization and polyploidy on adaptation and diversification. Additionally, population genetic considerations are relevant, especially the extremely reduced effective population sizes that are involved in the early stages of polyploid formation and stabilization in many groups. These conditions minimize the importance of selection relative to drift, thus further facilitating the survival of less than perfectly adapted genomes and genotypes while highlighting the role of “chance” or stochasticity (Lynch, 2007) in generating genome complexity as well as biological diversity.

Predicting long-term effects of phenomena that have profound impact on organisms, and whose evolutionary fate is heavily dependent on spatio-temporal contexts, is not yet possible. Even retrospectively constructing the detailed history of these phenomena in current lineages is complicated and this has fueled conflicting views (Mayrose et al., 2011; Abbott et al., 2013; Butlin and Ritchie, 2013; Soltis P. S. et al., 2014). The evolutionary consequences of allopolyploidy and hybridization—particularly over the longer term—have been and will remain a matter of interest into the future. Yet we see promise in our growing understanding of biological processes ranging in scale from the molecular to the ecological. This enhanced understanding of the genotype-to-phenotype equation should increasingly inform comparative and ecological analyses of adaptation, thus permitting an improved appreciation of the temporal dynamics and genomic underpinnings of polyploidy-fueled diversification.

## Author Contributions

All authors listed have made a substantial, direct and intellectual contribution to the work, and approved it for publication.

## Conflict of Interest

The authors declare that the research was conducted in the absence of any commercial or financial relationships that could be construed as a potential conflict of interest.
